# Cluster analysis for identifying sub-groups and selecting potential discriminatory variables in human encephalitis

**DOI:** 10.1186/1471-2334-10-364

**Published:** 2010-12-31

**Authors:** Jemila S Hamid, Christopher Meaney, Natasha S Crowcroft, Julia Granerod, Joseph Beyene

**Affiliations:** 1 Surveillance and Epidemiology, Ontario Agency for Health Protection and Promotion, Toronto, Canada; 2 Dalla Lana School of Public Health, University of Toronto, Toronto, Canada; 3 Department of Family and Community Medicine, University of Toronto, Toronto, Canada; 4 Health Protection Agency, Centre for Infections, London, UK; 5 Population Genomics Program, Department of Clinical Epidemiology and Biostatistics, McMaster University, Hamilton, Canada

## Abstract

**Background:**

Encephalitis is an acute clinical syndrome of the central nervous system (CNS), often associated with fatal outcome or permanent damage, including cognitive and behavioural impairment, affective disorders and epileptic seizures. Infection of the central nervous system is considered to be a major cause of encephalitis and more than 100 different pathogens have been recognized as causative agents. However, a large proportion of cases have unknown disease etiology.

**Methods:**

We perform hierarchical cluster analysis on a multicenter England encephalitis data set with the aim of identifying sub-groups in human encephalitis. We use the simple matching similarity measure which is appropriate for binary data sets and performed variable selection using cluster heatmaps. We also use heatmaps to visually assess underlying patterns in the data, identify the main clinical and laboratory features and identify potential risk factors associated with encephalitis.

**Results:**

Our results identified fever, personality and behavioural change, headache and lethargy as the main characteristics of encephalitis. Diagnostic variables such as brain scan and measurements from cerebrospinal fluids are also identified as main indicators of encephalitis. Our analysis revealed six major clusters in the England encephalitis data set. However, marked within-cluster heterogeneity is observed in some of the big clusters indicating possible sub-groups. Overall, the results show that patients are clustered according to symptom and diagnostic variables rather than causal agents. Exposure variables such as recent infection, sick person contact and animal contact have been identified as potential risk factors.

**Conclusions:**

It is in general assumed and is a common practice to group encephalitis cases according to disease etiology. However, our results indicate that patients are clustered with respect to mainly symptom and diagnostic variables rather than causal agents. These similarities and/or differences with respect to symptom and diagnostic measurements might be attributed to host factors. The idea that characteristics of the host may be more important than the pathogen is also consistent with the observation that for some causes, such as herpes simplex virus (HSV), encephalitis is a rare outcome of a common infection.

## Background

Encephalitis is an acute clinical syndrome of the central nervous system (CNS), often associated with fatal outcome or permanent damage, including cognitive and behavioural impairment, affective disorders and epileptic seizures [1]. It is a rare disease with annual incidence ranging between 3.5 and 7.4 cases per 100,000 persons, worldwide [2]. It is more common in children, the elderly and people with a weakened immune system (e.g. those with HIV/AIDS or cancer) [1-3]. For example, paediatric encephalitis rates in the UK have been estimated at 16 cases per 100,000 individuals per year [2]. In the UK, the most recent data estimate an annual rate of 1.5/100,000 in the general population and 2.8/100,000 in children, with the highest incidence in infants under the age of one (8.7/100,000) [1,3]. Encephalitis affects both sexes; however, it appears in slightly higher rates in males [1,4,5]. Although a rare condition, encephalitis has been recognized worldwide as an important public health issue because of the high morbidity and mortality associated with the disease and the potential for high public concern where new agents appear or spread into new population centres [1,6].

To date, infection of the CNS is considered to be the major cause of encephalitis and more than 100 different pathogens have been recognized as causative agents [1]. However, an estimated 32-85% of cases have unknown disease etiology [1,3,5-9]. The percentage of cases of unknown etiology varies greatly and is linked to the quality of microbiological laboratories. Other possible reasons include samples being taking at wrong time and possibility of new and emerging infections. For instance, about 85% of the 189 cases in a study conducted in Minnesota, USA are of unknown cause [9]. In another study conducted in California, about 65% of the 334 cases are of unknown etiology [8]. In a study conducted in the UK, about 60% of 700 cases are of unknown etiology [3]. Among the known causes, herpes simplex virus (HSV) has been recognized worldwide as the most common etiology [1,10]. However, most common etiologies differ from one territory to another depending on local epidemiological and ecological conditions. In some European countries, for instance, tick-borne encephalitis is the most prevalent etiology. The main infectious causes of encephalitis are listed in a review paper by Granerod and Crowcroft [1]. Possible risk factors associated with the disease have also been identified [1,11].

Although encephalitis has been studied extensively, there have not been any comparisons made among these studies mainly because there have not been any standard statistical methods used to describe and analyze the encephalitis data sets. To our knowledge, all studies have used descriptive statistics such as proportion and average as means of presenting their findings. Moreover, many studies considered groups according to causal agents and focused on a particular type of encephalitis with a specific etiology for instance. No formal statistical analysis has been performed to explore the data and identify possible clusters inherent in the encephalitis data sets.

The main objective of this paper, therefore, is to perform exploratory cluster analysis using the England human encephalitis data set with the aim of achieving a better understanding of human encephalitis and generate hypotheses about etiology. In particular, we aim to: 1) identify noise variables that have little or no contribution and filter out these variables from the data; 2) identify or determine subgroups of encephalitis; 3) identify major clinical and laboratory features/characteristics associated with encephalitis, and determine which of these variables distinguish a particular cluster from others; 4) identify major risk factors associated with encephalitis.

We use complete linkage hierarchical clustering with a simple matching distance matrix which is appropriate for binary data. We performed two dimensional clustering where patients and variables are clustered simultaneously. We use a heatmap to graphically display and visually assess underlying patterns that may exist in our data set. A cluster heatmap is one of the most widely used tools in biological sciences [12,13]. Weinstein describes cluster heatmaps as the most popular graphical representation which compacts large amount of information into a small space to bring out coherent patterns in the data [14]. Despite their widespread application in biological sciences, their application has been limited in epidemiology. To our knowledge, this is the first time that heatmaps have been used in this way. In addition to the main objectives stated above, this article, introduces heatmaps to clinical and epidemiological research in general and the study of encephalitis in particular.

## Methods

### Data Description

Our data relate to a prospective multicenter study where 268 patients are recruited from 24 hospitals/neurological centres in three geographical locations (South West, London, North West) across England. Appropriate ethical approval has been obtained and written informed consent from all patients or from their next of kin was received for the study. The manuscript has also been approved by the UK department of health. Details about the ethical approval can be found in the original study [15]. Variables used in the analysis include 1) symptom variables such as fever, lethargy and confusion 2) exposure variables such as water exposure, tick and mosquito bites 3) diagnostic/laboratory measurements from brain scans and measurements from cerebrospinal fluids. Most of the variables in the study are binary indicating presence and absence of attributes. Variables that are not binary have been dichotomized in the cluster analysis. Demographic variables such as sex and age as well as other clinical features including length of stay in the hospital and duration of illness are also provided in the data set. Variables used in our study are listed in Table 1.

**Table 1:** Variables included in our study

Symptom Variables	Exposure Variables	Diagnostic Variables	Demograhic Variables
• Lethargy	• Animal contact	• Measurements from Cerebrospinal fluid (CSF)	• Gender
• Personality/behavioural change	• Tick bite	- White blood cell count	• Age
• Seizures	• Mosquito bite	- Protein	• Region
• Stiff neck	• Insect bite	- Glucose	
• Headache	• Immunization	• Magnetic resonance Imaging (MRI)	
• Irritability	• Recent infection	• Electroencephalography (EEG)	
• Fever	• Travel abroad	• Computed Tomography (CT) scans	
• Focal neurological findings	• Travel within UK	• other clinical variables	
• Coma	• Raw fish	- Length of stay in hospital	
• Neurological signs	• Untreated water	- Duration of illness	
• Gastrointestinal	• Head trauma		
• Respiratory	• Sick person contact		
• Confusion	• Water exposure		
• Photophobia			
• Rash			
• Urinary			

### Methods

Exploratory cluster analysis is implemented with the aim of identifying sub-groups in human encephalitis. We also use patient information to perform clustering of variables to identify sets of variables that are clustered in some way. This allows us to explore similarities and differences between the groups of variables presented in Table 1. Cluster analysis is an exploratory statistical technique used for identifying patterns or groups in a data set where individuals within a given group are similar with respect to a specific similarity matrix. We use the heatmap for displaying clustering of patients and variables simultaneously. The heatmap also allows us to visually identify noise variables that do not discriminate between patients. In this paper, we follow three crucial steps in performing exploratory statistical analysis. The three steps are: variable/feature selection, choice of similarity measure and choice of clustering technique.

### Variable Selection

Variable or feature selection is an important tool in any clustering or classification problem, in particular when studies involve large number of variables. Variable selection helps in reducing the dimensionality of data and facilitating data visualization and understanding. Moreover, some of the variables might add noise while providing little or no information in identifying the underlying pattern inherent in the data set. In fact, it has previously been indicated that presence of noise variables may mask underlying pattern or structure in the data set [16]. Initial screening of variables is, therefore, a crucial step in any pattern identification task. Variable selection in the context of cluster analysis has been investigated by many, and several methods, including automatic selection, have been proposed [16,17]. Here we use a simple and intuitive approach by visually assessing the heatmap to see if a given variable has the same value for most of the individuals and hence is non informative in discriminating. We also supplement this graphical/visual assessment by calculating the proportion at which a given variable is present or absent. We exclude variables that are similar for more than 80% of patients. Variables for which a significant number of values are missing have also been removed from the analysis.

### Choice of Similarity Matrix

A fundamental step when performing cluster analysis is choosing an appropriate similarity matrix that quantifies how similar individuals are with respect to measurements provided in the variables. The most commonly used similarity measure is Euclidian distance. However, Euclidian distance may not be appropriate when the data consists of binary variables. There are a few similarity measures proposed for binary data. In this paper, we focus on the Sokal and Michener's distance matrix which is based on simple matching and gives equal weight for presence and absence [18]. For comparison purposes, however, we perform the analysis using the Jaccard and Euclidian distances as well [19,20].

Suppose we have two binary sequences, X_i _and X_j_, consisting of presence (+) or absence (-) of attributes. This results in the 2 × 2 association table shown in Table 2 below. Many distance matrices or similarity coefficients have been derived from such an association table [19,20]. Here we will present the two commonly used distance/similarity measures for binary data: Jaccard and simple matching similarity measures. Other similarity matrices are discussed in the papers by Gower and Gower and Legendre [21,22].

**Table 2:** Number of agreements or disagreements between two individuals based on a binary variable consisting of presence or absence of a particular attribute.

		Individual i		
		Present (+)	Absent (-)	Totals
Individual j	Present (+) Absent (-)	*a* *c*	*b* *d*	*a+b* *c+d*

	Totals	*a+c*	*b+d*	*Ν*

#### Jaccard's Distance

The Jaccard similarity matrix is the most commonly used distance matrix for binary data. It is defined as [18,19]

$$D_{ij}=\frac{b+c}{a+b+c}$$

Similarly, one can use the Jaccard similarity coefficient which is defined as *(1-D_i, j_) *and is given by

$$S_{ij}=\frac{a}{a+b+c}$$

#### Simple Matching (SM) distance

Binary data can be nominal or ordinal where in the latter case presence ("1") more in some sense than absence ("0"). It is, therefore, important to make distinction between similarity coefficients that do or do not incorporate *d *[23]. If data are nominal, coefficients for which the quantities *a *and *d *are equally weighted are more appropriate [20,23]. One such distance matrix, among others, is the Simple Matching (SM) distance due to Sokal and Michener [18]. It is defined as

$$D_{ij}=\frac{b+c}{a+b+c+d}$$

The corresponding similarity coefficient is

$$S_{ij}=1-D_{ij}=\frac{a+d}{a+b+c+d}$$

### Clustering Method

Clustering is a widely used exploratory and hypothesis generating approach in biological studies and has played important roles in identifying subtypes in complex diseases. One successful application, for instance, is in discovering cancer subtypes using clinical, gene expression and other types of genomic, clinical and epidemiological data. Here we use the hierarchical agglomerative approach; the most commonly used clustering approach, which has been demonstrated to be a useful tool in discovering sub-structures inherent in a given data set. Hierarchical agglomerative algorithms start with n observations and join individuals hierarchically where individuals with smallest distance apart join first. At each stage, the distance matrix is re-computed according to a linkage function. There are different hierarchical approaches depending on the type of the link function. Here we use the complete linkage or the furthest neighbour approach where the distance between two clusters is computed as the maximum of individual -to -individual distances, that is

$$L(C_{i},C_{j})=\underset{x\in C_{i},y\in C_{j}}{\max}d(x,y),$$

where *d *is the distance function, and *C_i _*and *C_j _*are two sets of clusters.

## Results

Our data set consists of 268 patients; among them 209 patients are confirmed encephalitis cases. These patients are included in subsequent analyses. Further, demographic variables (age, sex, region) as well some clinical variables (duration of illness and length of stay) in the hospital, are removed when performing cluster analysis. However, they are used in describing the clusters and assessing whether an age, gender, or region stratified clustering is appropriate for our data set.

Table 3 shows summary statistics of our data set. The result show that men are at a slightly higher (54.5%) risk of encephalitis than women (45.5%). Most of the encephalitis patients are children and young adults (mean age = 33) where a large proportion of the patients are children of age ≤ 10 (26%) indicating that young children are at higher risk of developing encephalitis. Our results also show that 84 (40%) of the 209 encephalitis cases are of unknown etiology. Among patients with known etiology, herpes simplex encephalitis is the most common etiology and the next common etiology is Acute Disseminated Encephalitis (auto-immune induced encephalitis) (Table 3).

**Table 3:** Descriptive statistics on selected demographic and clinical variables for the 209 encephalitis patients

Variable	Group	N	%
Aeitiology	HSV^a^	38	18.18
	ADEM^b^	23	11
	VZV^c^	12	5.74
	MTB^d^	10	4.78
	ANT^e^	8	3.83
	Other	34	16.27
	Viral	22	10.53
	Bacterial	12	5.74
	Unknown	84	40.19
			
Gender	Male	114	54.54
			
Age: Categorical	Age ≤ 10	55	26.32
	10 < Age ≤35	55	26.32
	35 < Age ≤55	42	20.1
	Age >55	50	23.92
			
Age: Continuous	33.29 ± 25.64 (0,87)*		
			
Region	South West	17	8.13
	London	86	41.15
	North West	106	50.72
			
Duration of illness	58.88 ± 71.39 (3,535)*		
			
Length of stay**	49.01 ± 67.01(2,521)*		

** Length of stay in the hospital

^a ^Herpes Simplex Virus

^b ^Acute Disseminated Encephalitis (auto-immune induced)

^c ^Varicella Zoster Virus

^d ^Mycobacterium Tuberculosis

^e ^Other immune-mediated encephalitis

^* ^mean ± sd (min, max)

Some of the variables have no or little missing values. The symptom variables, for instance, have no missing information whereas the exposure variables seem to have between 6-8% missing values. However, exposure variables appear to be very sparse in the sense that most of these variables are absent for the majority of the patients. We will discuss this in the variable selection step. There is a considerable percentage of missing values in the two brain scan variables (16.7% and 17.7% missing values for CT and MRI, respectively) and a high percentage of missing values (42.6%) was observed in the EEG variable. Further description of data including case definition as well as descriptive analysis of the dataset is presented in Granerod et al. [15].

### Variable selection

**Figure 1:**
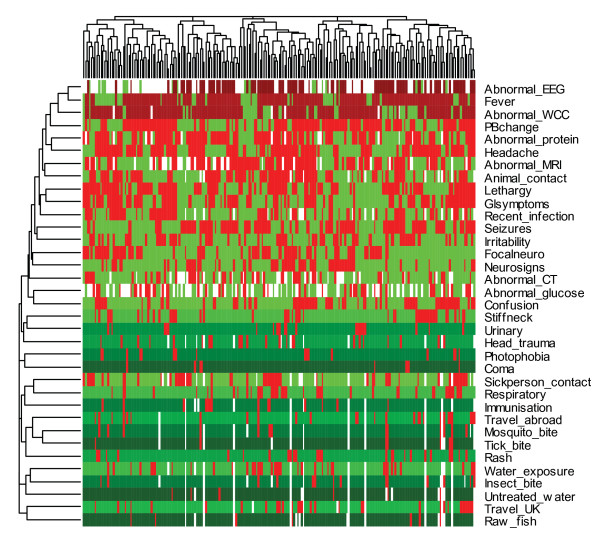
**Heatmap showing the standardized 209 by 35 dimensional matrix of encephalitis data set where row wise standardization is performed by subtracting the mean and dividing it by its standard deviation**.

A heatmap showing two dimensional clustering of variables and patients for our data set is displayed in Figure 1 where cluster relationships in the form of dendograms are indicated on the top (representing patient clustering) and on the left (representing clustering of variables). A total of 209 encephalitis patients and 35 variables (shown in Figure 1) are included in our initial analysis although some are filtered during the variable selection step. Complete linkage hierarchical clustering with the simple matching distance matrix is used in constructing the heatmap. However, similar results were also obtained the Euclidean and Jaccard distance matrices. The 209 by 35 dimensional matrix of encephalitis data set is displayed on the heatmap where each column represents the standardized binary measurements for a given patient. Presence of a particular attribute is coded as red and its absence coded as green. The intensities of red and green colours, therefore, represent the proportion of presence and absence of a particular attribute for a given patient. Missing values are represented as white in the heatmap. Coherent patterns of colour are generated by hierarchical clustering on both horizontal and vertical axes to bring like together with like.

Among the symptom and diagnostic variables, for instance, fever is present in more than 78 percent of the patients and hence mostly represented by dark red colour. Although there are a significant number of missing values in the EEG and WCC variables, the percentage of presence in both is much higher than absence and hence results in a row represented mostly by a dark red colour. Coma and stiff neck, on the other hand, are represented by dark green colours indicating that these symptoms are absent in most of the encephalitis patients. Looking at the distribution of colour in the heatmap, therefore, helps us in identifying informative variables in our data set.

Figure 1 also shows that variables on the bottom part of the histogram are sparse (mostly zero) and hence are not helpful in identifying subgroups in our encephalitis data set. This part of the heatmap mostly consists of exposure variables indicating that these variables might not be helpful in identifying sub-groups of encephalitis. However, some the exposure variables, represented by a combination of light green and light red colours, might be risk factors associated with encephalitis. Water exposure and travel in the UK, for example, are two among them. Travel outside the UK seems to be associated with encephalitis as well but did not meet the variable selection criteria (present for more than 80% of the patients) and hence is removed from the cluster analysis. It is, however, important to mention that a careful investigation through association analysis should be conducted before ruling such variables out from being possible risk factors. Variables on the top of the heatmap, however, seem very informative. The majority of these variables are symptom and laboratory variables. Among the exposure variables, animal contact, recent infection, and sick person contact are also shown to be informative. These variables have previously been identified as possible risk factors associated with encephalitis. Symptom variables such as coma and photophobia do not appear to help discriminate between sub-groups of encephalitis. Moreover, our data set seems to indicate that these symptom variables are not a common characteristic of encephalitis.

**Figure 2:**
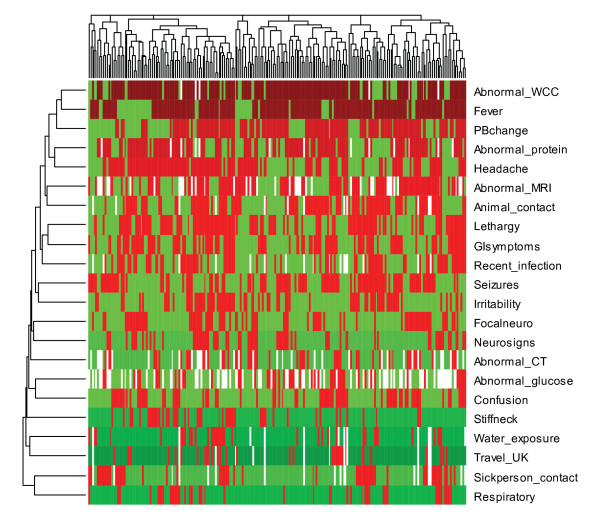
**Heatmap representing cluster of patients and variables after filtering out variables that are zero (absent) in more than 80% of the 209 encephalitis patients**.

We removed 12 variables that are absent (present) for more than 80% of encephalitis patients. Moreover, electroencephalography (EEG) is also removed from the analysis because of high percentage (about 43%) missing values. Furthermore, more than 90% of the patients have an abnormal EEG and hence not helpful in discriminating among patients. The variable indicating an abnormal glucose level in cerebrospinal fluid also contains a significant percentage of missing values (37%). However, we decided to keep this variable in our analysis because the variable, when measured, is evenly distributed among the encephalitis patients and hence might be helpful in distinguishing some of the clusters. A heatmap, constructed using the remaining 22 variables, is displayed in Figure 2. Except for a small percentage of sparseness displayed in the very bottom of the heatmap, it can be seen from the figure that most of the variables seem to be informative. Figure 2 indicates that animal contact and recent infection might be potential risk markers associated with encephalitis. Moreover, water exposure and sick person contact are also indicated as potential risk factors. The dark red colour in the heatmap indicates that fever and abnormal white blood cell counts (WCC) are indicated as the two major characteristics of human encephalitis. The slightly lighter red colour in heatmap also shows that headache, abnormal protein in CSF and personality and behavioural changes are observed in the majority of encephalitis patients. The amount of white in the row representing glucose level shows the high amount of missing values mentioned earlier.

### Cluster Analysis

Clustering of our encephalitis data set using agglomerative hierarchical clustering approach revealed six major clusters. An additional small cluster, consisting of 5 encephalitis patients, was also observed. All of these 5 patients had seizures. Abnormal EEG and fever are observed in 4 (80%) of the patients. No other symptom was observed for more than one patient; moreover, most of the exposure variables are zero for these patients. The only exception is water exposure where 3 out of the 5 patients were exposed to untreated water. The dendogram from the analysis is displayed on the top of the heatmap shown in Figure 2. The maximum distance is used as a cut-off point indicating that these are the minimum number of groups observed in our data set. However, it can be seen from the dendogram that there are three relatively large clusters that can be divided further into more homogenous sub-groups by choosing a slightly smaller cut-off point.

The main characteristics and other features that distinguish the 6 major clusters are presented in Table 4. A given characteristic is considered as the main characteristic of a cluster if the variable coding this attribute is present (or absent) for more than 80% of the patients in a particular cluster. If it is one (present) for more than 50% of the patients (but less than 80%), it is listed under other characteristics in Table 4. Cluster 1, for instance, is characterized by abnormal WCC, lethargy, headache and fever where these symptoms were observed in more than 80% of the patients. Abnormal protein, personality and behavioural change as well as gastro-intestinal symptoms were also observed in a significant percentage of patients in this group. A considerable proportion of patients in this cluster had also had animal contact.

**Table 4:** Description of the 6 major clusters identified for the UK encephalitis data using hierarchical clustering.

Cluster	Major Characteristics	Other
Cluster 1 (n = 61)	Abnormal WCC, lethargy, headache, fever	Abnormal protein, gastro-intestinal symptoms, personality and behavioural change, animal contact
Cluster 2 (n = 50)	Abnormal protein, WCC, personality and behavioural change, fever	Animal contact
Cluster 3 (n = 44)	Personality and behavioural change, fever,	Abnormal MRI, abnormal EEG, abnormal protein, abnormal WCC, lethargy, headache, gastro-intestinal animal contact
Cluster 4 (n = 21)	Lethargy, Seizure, fever, animal contact	Abnormal MRI, abnormal EEG, abnormal WCC, irritability, personality and behavioural change, recent infection
Cluster 5 (n = 15)	Abnormal EEG, abnormal WCC, seizure, headache, sick person contact	Fever
Cluster 6 (n = 13)	Abnormal MRI, headache	Abnormal CT, abnormal protein, abnormal WCC, lethargy, personality and behavioural change, focal neurological abnormality, other neurological symptoms

The dendogram revealed marked within cluster heterogeneity and subgroups within Cluster 1. Further investigation of this cluster to identify more homogenous sub-clusters might, therefore, be of interest. With a slightly lower cut-off point, Cluster 1 can be subdivided into two sub-clusters. The first sub-group is characterized by abnormal WCC, lethargy, headache and fever; whereas, the major characteristics of the second sub-group are abnormal protein, abnormal WCC, lethargy, headache and gastro-intestinal symptoms. It is important to note that focal neurological abnormalities were observed in all of the patients in the second sub-cluster whereas only a small proportion of patients in the first sub-cluster had this symptom. Animal contact was identified as a major characteristic of sub-group 2, although only a small proportion of patients in the first sub-group were exposed to this risk factor. Another important difference between the two sub-clusters is fever. Although fever is the main characteristic of cluster 1, it was not observed in any of the patients in the second sub-group. This is an important finding since fever is considered one of the main characteristics of encephalitis. Further investigation of this sub-cluster is required in understanding the nature of encephalitis for these particular patients. Irritability, personality and behavioural change, stiff neck, gastro-intestinal symptoms were observed in some of the patients in sub-group 1; whereas, confusion was observed in a significant proportion of patients in sub-group 2. The dendogram also reveals that the first sub-cluster can further be divided into two more homogenous groups. We investigated these subgroups further and learned that one of them was characterized by irritability and personality and behavioural change whereas the major characteristic for the other subgroup is abnormal CSF.

The major characteristics common to patients in Cluster 2 are abnormal protein, abnormal WCC, personality/behavioural change and fever. A significant proportion of patients in this cluster (about 50%) had animal contact. The dendogram also reveals two more homogenous sub-groups of Cluster 2. One of the sub-groups is characterized by abnormal protein whereas patients in the second subgroup showed personality and behavioural changes.

The third largest cluster consists of 44 encephalitis patients and is characterized by fever and personality/behavioural change. A significant proportion of these patients show many other symptoms (Table 4) and had abnormal brain scans and laboratory measurements. In comparison to the rest of the clusters, patients in Cluster 3 showed more symptoms. Only two symptoms, fever and personality/behavioural change, were identified as the two major characteristics. Further investigation of this cluster reveals that patients in this cluster have been ill for very long time and stayed in the hospital (the maximum duration of illness and hospital stay was observed in this cluster). Cluster 3 can also be subdivided into two more homogeneous subgroups, one characterized by abnormal protein and WCC, and another characterized by personality and behavioural change (where more than 94% of the patients in this sub-group showed this symptom). Although fever is present in more than 75% of the patients in the encephalitis data set, it is the major characteristic for some of the clusters and is not so for others.

Overall, symptom variables are identified as important variables in distinguishing the clusters. However, animal contact, sick person contact and recent infection have been identified as major characteristics for some of the clusters. These exposure variables have also been observed in a large proportion of patients in some of the clusters.

A slightly higher proportion of males were observed in our data set. Overall, similar age distribution was also observed in the six major clusters suggesting that age stratified clusters or subgroups might not be inherent in the data. Similarly, the data does not provide any indication of stratified clustering with respect to region. On average, the duration of illness was longer for patients in Clusters 2 and 5 compared to the other clusters. These patients also had longer hospital stays. The average duration of illness for patients in Cluster 4, however, is much smaller than the rest of the clusters. These patients, on average, spent less time in the hospital.

**Figure 3:**
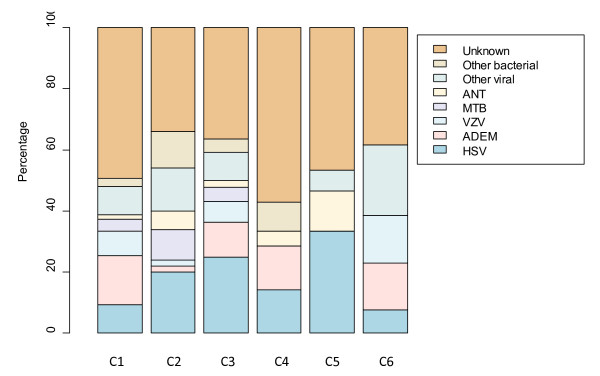
**Distribution of etiological groups for the 6 major clusters identified for the encephalitis data set, where C1 to C6 represent the six identified clusters**.

In general, our results show that each of the clusters consist of patients from all the etiological groups where the majority of encephalitis patients in each of the clusters are of unknown cause. The distribution of the etiology groups in the six major clusters is provided in Figure 3. This suggests that encephalitis cases, although caused by different etiological agents, do not in general cluster according to agent related etiology. Rather, the results suggest that the patients are clustered according to symptom and laboratory variables. This would be consistent with host factors rather than agent factors being important for determining outcome.

## Discussion and Conclusions

Hierarchical cluster analysis revealed six major clusters in the England encephalitis data set. An additional small cluster consisting of 5 patients was also observed in the data. It is in general assumed and is common practice to group encephalitis cases according to disease etiology. However, our results indicate that patients are clustered with respect to mainly symptom and laboratory variables rather than causal agents. In fact, disease etiology is distributed across all clusters where the majority of the patients in all the clusters are of unknown etiology. There is some intuitive sense to the clustering which deserves further exploration in relation to clusters of symptoms and laboratory variables which seem to exclude certain causes. Tuberculosis (MTB) only appears in 3 of the 6 clusters; varicella zoster virus (VZV) does not appear in clusters 4 or 5; "other viral" not in 4; acute disseminated encephalomyelitis (ADEM) is not in 5; other bacterial not in 5 or 6; and antibody-mediated encephalitis (ANT) is not found in cluster 6. Furthermore, exposure variables appear to be non informative towards discriminating among the clusters. These similarities and/or differences with respect to symptom and laboratory measurements might, therefore, be attributed to other factors that are not included in our data set. For instance, life style and other environmental factors not accounted for in our data set might play a role, although control data were not available for comparison. Another possible explanation could be genetic variation between the cases. Although genetics have not been shown to be associated with encephalitis, it has not been ruled out in any of the studies and it would be worth investigating whether it plays a role in disease prognosis. The idea that characteristics of the host may be more important than the pathogen is also consistent with the observation that for some causes, such as herpes simplex virus (HSV), encephalitis is a rare outcome of a common infection. Our results also show that there is a considerable heterogeneity within the clusters and each of the clusters can be divided further to more homogenous subgroups. Further investigation is, required to have a better understanding of the groups identified in our cluster analysis.

We have also done variable clustering that can be helpful in identifying risk factors, developing prognostic models and improving existing diagnosis and detection, and evaluating new laboratory diagnostic methods. Our study confirmed symptom variables, such as fever, personality and behavioural change, headache and lethargy, as the main characteristics of encephalitis. Laboratory variables such as brain scan and measurements from cerebrospinal fluids are also identified as indicators of encephalitis. This suggests that symptom variables can be used for improving existing diagnosis as well as developing new diagnostic algorithms. The distribution of the etiology groups across the clusters suggests that the patients are clustered according to symptom and laboratory variables. This would be consistent with host factors rather than agent factors being important for determining outcome. For instance, infection of HSV is very common; however, most HSV infections do not lead to encephalitis. This indicates that factors other than agents play important roles in developing encephalitis from HSV infection.

Among the exposure variables, however, animal contact and exposure to recent infection have only been identified as possible risk factors. Exposure to untreated water and sick person contact are also linked to encephalitis suggesting that these variables might be potential risk factors. However, further statistical analysis focused more on variable selection is required to identify potential risk factors. This might help researchers to devise new preventive measures. It might also be interesting to study the association between the different sets of variables in our data set to identify an optimal combination of variables that would be helpful in developing a predictive model.

Our data set is sparse in nature. Consequently, some of the variables might be more of a noise, and as a result might mask the underlying substructure inherent in the data. In this manuscript, we filtered out some of the variables using a heatmap along with an ad hoc cut-off point. It might, however, be interesting to incorporate an automatic variable selection that takes the sparseness into account in the cluster analysis. Cluster analysis is exploratory in nature and our results are a first step in understanding the structure of the data from the UK Encephalitis Study. It can also be viewed as a hypothesis generating approach. Even though our cohort is one of the largest of its kind, a small sample size is one of the limitations of this study because the statistical analysis involved multivariate modelling with large number of variables. Encephalitis is a rare disease and most of the previous studies are mainly based on a much smaller number of patients. We are currently exploring extensions to the analysis such as estimating the optimal number of clusters in the data set and cluster reliability through cluster stability scores. We believe this is the first application of cluster analysis to an epidemiological study of encephalitis, and a novel approach to simultaneously displaying the distribution of variables and the clustering of cases and variables, and guiding variable selection. It has generated hypotheses about etiology and indicated where focus needs to placed in future analyses. One of the challenges of encephalitis is that agents are not found in most of the patients where up to 60%-85% of patients are of unknown etiology. This is because most infections of the central nervous system (CNS) are secondary infections, consequently, exposure factors (such as previous infection, sick person contact, animal contact as well as insect bite) are important to understand etiology and epidemiology of encephalitis. Our results show that cases of unknown etiology are distributed throughout the six clusters. Clinicians might understand disease etiology and provide better diagnostics of these patients by investigating other patients with known etiology in the same cluster. In conclusion, we would like to mention that t identifying infectious agents is important to control infectious encephalitis and most studies in the literature have used this approach. However, as treatment is available for only some types of encephalitis and the cause is unknown in most cases, we adopted a novel approach to look at clinical symptoms and exposure factors irrespective of etiology. We believe this method could also be helpfully applied to other syndromes for which a cause is often not found, such as acute respiratory and gastrointestinal disease, and may have numerous other applications in epidemiology.

## Competing interests

The authors declare that they have no competing interests.

## Authors' contributions

JSH contributed to the conception of study and statistical methods, performed analysis and interpretation of data, and drafted the manuscript. CM contributed to the statistical methods, analysis and interpretation of data, and participated in drafting the manuscript. NSC and JG contributed to the conception of the study, acquisition of data and helped with critical revision of the manuscript for important intellectual content. JB contributed to the conception of the study, statistical methods, interpretation of data, and participated in drafting the manuscript. All authors have read and approved the final version of the manuscript.

## Pre-publication history

The pre-publication history for this paper can be accessed here:

http://www.biomedcentral.com/1471-2334/10/364/prepub
